# The Impacts of Systemic Immune-Inflammation Index on Clinical Outcomes in Gallbladder Carcinoma

**DOI:** 10.3389/fonc.2020.554521

**Published:** 2020-10-23

**Authors:** Lejia Sun, Yukai Jin, Wenmo Hu, Mengyuan Zhang, Bao Jin, Haifeng Xu, Shunda Du, Yiyao Xu, Haitao Zhao, Xin Lu, Xinting Sang, Shouxian Zhong, Huayu Yang, Yilei Mao

**Affiliations:** ^1^Department of Liver Surgery, Peking Union Medical College (PUMC) Hospital, PUMC & Chinese Academy of Medical Sciences, Beijing, China; ^2^Peking Union Medical College (PUMC), PUMC & Chinese Academy of Medical Sciences, Beijing, China

**Keywords:** gallbladder carcinoma, prognosis, systematic inflammation markers, systemic immune-inflammation index, nomogram

## Abstract

**Background:** Systemic immune-inflammation index (SII) is considered to be a prognostic marker in several cancers. However, the prognostic value of baseline pre-operative SII in gallbladder carcinoma (GBC) has not been evaluated. This study aimed to determine the prognostic significance of SII and generate a predictive nomogram.

**Methods:** We retrospectively studied 142 GBC patients who underwent surgical resection at the Peking Union Medical College Hospital between 2003 and 2017. SII, neutrophil-to-lymphocyte ratio (NLR), and lymphocyte-to-monocyte ratio (LMR) were evaluated for their prognostic values. A multivariate Cox proportional hazards model was used for the recognition of significant factors. Then, the cohort was randomly divided into the training and the validation set. A nomogram was constructed using SII and other selected indicators in the training set. C-index, calibration plots, and decision curve analysis were performed to assess the nomogram's clinical utility in both the training and the validation set.

**Results:** The predictive accuracy of SII (Harrell's concordance index [C-index]: 0.624), NLR (C-index: 0.626), and LMR (C-index: 0.622) was evaluated. The multivariate Cox model showed that SII was a superior independent predictor than NLR and LMR. SII level (≥600) (hazard ratio [HR]: 1.694, 95% confidence interval [CI]: 1.069–2.684, *p* = 0.024), carbohydrate antigen (CA) 19-9 level (≥37 U/ml) (HR: 2.407, 95% CI: 1.472–3.933, *p* < 0.001), and TNM stage (*p* = 0.026) were selected to construct a nomogram for predicting overall survival (OS). The predictive ability of this model was assessed by C-index (0.755 in the training set, 0.754 in the validation set). Good performance was demonstrated by the calibration plot. A high net benefit was proven by decision curve analysis (DCA).

**Conclusion:** SII is an independent prognostic indicator in GBC patients after surgical resection, and the nomogram based on it is a useful tool for predicting OS.

## Introduction

Gallbladder carcinoma (GBC) is a rare neoplasm, but it is the most common type in the biliary tract. GBC accounts for 1.2% of cancer incidence and 1.7% of all cancer deaths worldwide and ranks as the sixth most common digestive tract cancer (1). The gallbladder is located beneath the liver, and this makes the early identification of neoplasms difficult. Besides, it is a lethal malignant disease and metastasizes rapidly (2). Most patients with GBC present with advanced-stage disease at diagnosis, and only <20% are eligible for curative surgical resection (3). This means that discrimination of patients at diagnosis is needed urgently for precise treatment decision-making. The American Joint Committee on Cancer (AJCC) staging system based on pathological characteristics is widely used for various cancers. However, it does not include patients' demographic, nutritional, and other clinical features. The immune system and inflammation play critical roles in neoplasia, which means that inflammatory markers, such as neutrophil-to-lymphocyte ratio (NLR), lymphocyte-to-monocyte ratio (LMR), platelet-to-lymphocyte ratio (PLR), and systemic immune-inflammation index (SII), could be used as prognostic factors. NLR has been widely used as a prognostic factor in pancreatic, lung, and gallbladder cancers and hepatocellular carcinoma (4–7). SII was first described for hepatocellular carcinoma and proved to be an independent predictor of poor survival of pancreatic ductal adenocarcinoma and gastric and colorectal cancers (8–12). However, most of these markers were evaluated separately, and no research analyzed the prognostic value of SII for GBC. This study retrospectively analyzed the prognostic significance of SII, NLR, and LMR and constructed a nomogram generated from these markers and other clinical features. This nomogram constructed a model for prediction of overall survival (OS) probability, which could eliminate the heterogeneity in the TNM staging system.

## Methods

### Patients

We retrospectively evaluated all patients who underwent surgical resection for GBC between January 2003 and January 2017 at the Peking Union Medical College (PUMC) Hospital. Patients were included if they underwent surgery for pathologically confirmed GBC. Patients without follow-up or available clinical data at the time of first diagnosis were excluded, as were patients with autoimmune diseases, other tumors, or recurrent tumors. This study was conducted in accordance with the Declaration of Helsinki and approved by the Ethics Committee of PUMC (number: S-K1110).

### Data Collection

Demographic, clinical, and laboratory data were retrospectively retrieved from the electronic medical records.

Demographic data included age at diagnosis, sex, and comorbidity. Comorbidity was scored by the age-adjusted Charlson Comorbidity Index (aCCI).

Pathological data including R0 resection rate, lymph node invasion rate, and AJCC stage (8th edition) were collected from surgical records and pathological reports.

Clinical outcomes evaluated were surgical blood loss, length of hospital stay, postoperative complications, postoperative length of hospital stay, and OS. Blood loss was estimated by surgeons. Other data were collected from medical records.

Laboratory data were obtained before surgery, including blood counts, carbohydrate antigen (CA) 19-9, serum albumin, and total bilirubin. The absolute platelet (P), neutrophil (N), lymphocyte (L), and monocyte (M) counts were used to calculate the following inflammatory biomarkers: neutrophil-to-lymphocyte ratio (NLR = N/L), lymphocyte-to-monocyte ratio (LMR = L/M), and systemic immune-inflammation index (SII = P × [N/L]).

### Statistical Analysis

Continuous data were shown as mean ± standard deviation or median [interquartile range (IQR)], whereas categorical variables were reported as frequency and percentage. For continuous variables, comparisons were made using Student's *t*-test or Mann–Whitney *U*-test as appropriate. Categorical variables were compared by chi square or Fisher's exact test. OS was calculated from the date of operation to the date of death. Kaplan–Meier analysis was used to estimate OS, and statistical significance for survival was determined by log-rank test. The cut-off values of SII, NLR, and LMR were determined by Harrell's C and Somers' D statistical tests, calculated by R package “Hmisc” (titled as Harrell Miscellaneous). The predictive accuracy of these markers was assessed by Harrell's concordance index (C-index) and time-dependent receiver operating characteristic (ROC) analyses. Univariate and multivariate Cox proportional hazard analyses were used to identify independent predictors of OS. Variables with a *p*-value no more than 0.1 in univariate analysis were then subjected to the multivariate analysis. The backward stepwise elimination method was used in building the multivariate models. Then, we randomly divided the whole cohort into the nomogram development set and validation set in a proportion of 1:1. A prognostic nomogram was established for predicting OS in the training cohort, and Harrell's C-index was used to measure the predictive accuracy in both the training and the validation cohort. We used the integrated discrimination improvement (IDI) index to evaluate the effect of a marker to the predictive ability of the model. Besides, IDI was also used to quantify the predictive ability of different prognostic models. Calibration plots and decision curve analysis (DCA) were used to assess predictive performance. All tests with a two-sided *p* < 0.05 were considered statistically significant. Statistical analysis was conducted with SPSS version 25 and R 3.6.3.

## Results

### Demographic and Pathological Characteristics

We identified 142 patients (82 female; 57.7%) with GBC for our analysis. The median age of the whole population was 63.06 years. The median CA 19-9 level was 45.7 U/ml (IQR, 12.8–220.9). Median SII was 595.4 (IQR, 373.6–1,089.5), median NLR was 2.57 (IQR, 1.73–4.04), and median LMR was 4.20 (IQR, 3.01–5.81). Median OS for our study was 21 months. Other clinicopathological data are summarized in Table 1.

**Table 1:** Demographic and pathological characteristics.

**Parameter**	**Total cohort (*n* = 142)**
Mean age (years)	63.06 ± 10.68
Sex (male)	60 (42.3%)
Median Alb (IQR) (g/L)	41 (37–43)
Median Tbil (IQR) (μmol/L)	12.80 (9.68–21.33)
Median NLR (IQR)	2.57 (1.73–4.04)
Median LMR (IQR)	4.20 (3.01–5.81)
Median SII (IQR)	595.4 (373.6–1,089.5)
Median CA 19-9 (IQR) (U/ml)	45.7 (12.8–220.9)
LNI	64 (45.1%)
R0 resection	88 (62.0%)
AJCC TNM stage	
0	5 (3.5%)
I	11 (7.7%)
IIA	12 (8.5%)
IIB	1 (0.7%)
IIIA	44 (31.0%)
IIIB	42 (29.6%)
IVA	9 (6.3%)
IVB	18 (12.7%)
Median OS (months)	21

*Alb, albumin; Tbil, total bilirubin; NLR, neutrophil-to-lymphocyte ratio; LMR, lymphocyte-to-monocyte ratio; SII, systemic immune-inflammation index; CA, carbohydrate antigen; LNI, lymph node invasion; AJCC, American Joint Committee on Cancer; OS, overall survival*.

### Association of Inflammatory Indicators and Baseline Characteristics

The cut-off points for the three inflammatory markers were 600 for SII, 2.5 for NLR, and 4.7 for LMR (Table 2).

**Table 2:** Inflammatory markers cut determination.

**SII cut**	**Cox HR (95% CI)**	***p*-value**	**Harrell's C**	**Somers' D**
400	2.252 (1.358–3.735)	0.002	0.600	0.199
500	2.251 (1.465–3.460)	<0.001	0.617	0.234
**600**	**2.261 (1.496–3.418)**	** <0.001**	**0.624**	**0.248**
650	2.270 (1.509–3.413)	<0.001	0.622	0.244
700	2.343 (1.559–3.520)	<0.001	0.624	0.248
750	2.220 (1.477–3.335)	<0.001	0.618	0.236
800	2.300 (1.529–3.460)	<0.001	0.618	0.236
900	2.214 (1.462–3.353)	<0.001	0.607	0.214
1,000	1.927 (1.249–2.972)	0.003	0.585	0.171
**NLR cut**	**Cox HR (95% CI)**	***p*****-value**	**Harrell's C**	**Somers' D**
2.00	2.368 (1.452–3.860)	<0.001	0.602	0.204
2.35	2.335 (1.519–3.489)	<0.001	0.621	0.241
2.40	2.410 (1.568–3.704)	<0.001	0.625	0.250
2.45	2.343 (1.530–3.589)	<0.001	0.623	0.246
**2.50**	**2.363 (1.551–3.600)**	** <0.001**	**0.626**	**0.252**
2.55	2.226 (1.471–3.368)	<0.001	0.623	0.247
2.60	2.156 (1.430–3.251)	<0.001	0.619	0.238
2.70	2.190 (1.445–3.293)	<0.001	0.620	0.240
2.75	2.176 (1.448–3.271)	<0.001	0.621	0.242
2.80	2.228 (1.483–3.347)	<0.001	0.625	0.251
2.85	2.206 (1.468–3.316)	<0.001	0.618	0237
2.90	2.206 (1.468–3.316)	<0.001	0.618	0.237
3.50	2.075 (1.373–3.137)	<0.001	0.606	0.211
**LMR cut**	**Cox HR (95% CI)**	***p*****-value**	**Harrell's C**	**Somers' D**
3.0	0.663 (0.423–1.037)	0.072	0.550	0.100
3.5	0.570 (0.379–0.856)	0.007	0.579	0.158
4.0	0.482 (0.321–0.726)	<0.001	0.605	0.210
4.3	0.454 (0.300–0.689)	<0.001	0.613	0.226
4.5	0.445 (0.290–0.680)	<0.001	0.617	0.233
4.6	0.445 (0.290–0.681)	<0.001	0.617	0.233
**4.7**	**0.422 (0.274–0.650)**	** <0.001**	**0.622**	**0.244**
4.8	0.454 (0.295–0.700)	<0.001	0.612	0.224
4.9	0.452 (0.292–0.699)	<0.001	0.612	0.225
5.0	0.482 (0.311–0.749)	0.001	0.603	0.206
5.5	0.466 (0.283–0.765)	0.003	0.587	0.173

*NLR, neutrophil-to-lymphocyte ratio; HR, hazard ratio; LMR, lymphocyte-to-monocyte ratio; SII, systemic immune-inflammation index; CI, confidence interval. Bold values represent the optimal cut off point whose C-index is the highest*.

The total cohort was divided into groups, and comparison between them is shown in Table 3. All three inflammatory markers were significantly correlated with albumin, CA 19-9, and other pathological characteristics (all *p* < 0.01). The SII-high group showed lower median albumin (39 vs. 42 g/L, *p* = 0.001), higher median CA 19-9 (100.3 vs. 22.8 U/ml, *p* = 0.009), more lymph node invasion (57.1 vs. 33.3%, *p* = 0.004), and lower R0 resection rate (50.0 vs. 73.6%, *p* = 0.004). No association was found between these inflammatory markers and age, sex, and total bilirubin.

**Table 3:** Comparison of baseline characteristics grouped by SII, NLR, and LMR.

**Parameter**	**SII**			**NLR**			**LMR**		
	** <600**	**≥600**	***p*-value**	** <2.5**	**≥2.5**	***p*-value**	** <4.7**	**≥4.7**	***p*-value**
	***N* = 72**	***N* = 70**		***N* = 67**	***N* = 75**		***N* = 81**	***N* = 61**	
Mean age (years)	64.22 ± 9.64	61.87 ± 11.60	0.191	63.69 ± 10.28	62.51 ± 11.06	0.513	63.15 ± 11.09	62.95 ± 10.20	0.914
Sex (male)	29 (40.3%)	31 (44.3%)	0.629	23 (34.3%)	37 (49.3%)	0.071	39 (48.1%)	21 (34.4%)	0.101
Median Alb (IQR) (g/L)	42 (39–45)	39 (35–42)	**0.001**	42 (38–45)	40 (36–42)	**0.007**	39 (36–43)	42 (39–45)	**0.003**
Median Tbil (IQR) (μmol/L)	11.70 (10.05–17.80)	15.25 (9.55–27.35)	0.070	11.40 (10.00–16.90)	15.20 (9.60–25.20)	0.052	14.70 (9.50–25.80)	11.40 (10.05–16.65)	0.080
Median NLR (IQR)	1.80 (1.45–2.33)	4.04 (2.98–5.20)	<0.001	1.72 (1.44–2.16)	3.86 (2.94–5.17)	<0.001	3.63 (2.72–4.97)	1.70 (1.43–2.14)	<0.001
Median LMR (IQR)	5.51 (4.26–6.47)	3.16 (2.30–4.06)	<0.001	5.83 (4.97–6.63)	3.17 (2.26–3.97)	<0.001	3.10 (2.24–3.87)	5.96 (5.36–7.28)	<0.001
Median SII (IQR)	375.5 (277.0–471.7)	1,092.0 (782.5–1,422.1)	<0.001	371.0 (273.3–473.5)	1,041.1 (650.2–1,385.1)	<0.001	956.1 (595.4–1,337.8)	374.5 (271.0–489.5)	<0.001
Median CA 19-9 (IQR) (U/ml)	22.8 (10.0–169.7)	100.3 (16.5–271.2)	**0.009**	21.7 (10.7–125.1)	106.0 (14.6–298.7)	**0.007**	98.1 (16.7–292.3)	21.4 (9.7–1,137.6)	**0.003**
LNI	24 (33.3%)	40 (57.1%)	**0.004**	20 (29.9%)	44 (58.7%)	**0.001**	48 (59.3%)	16 (26.2%)	** <0.001**
R0 resection	53 (73.6%)	35 (50.0%)	**0.004**	52 (77.6%)	36 (48.0%)	** <0.001**	38 (46.9%)	50 (82.0%)	** <0.001**
AJCC TNM stage			**0.002**			**0.001**			** <0.001**
0	4 (5.6%)	1 (1.4%)		4 (6.0%)	1 (1.3%)		1 (1.2%)	4 (6.6%)	
I	10 (13.9%)	1 (1.4%)		9 (13.4%)	2 (2.7%)		2 (2.5%)	9 (14.8%)	
IIA	7 (9.7%)	5 (7.1%)		7 (10.4%)	5 (6.7%)		4 (4.9%)	8 (13.1%)	
IIB	1 (1.4%)	0 (0.0%)		1 (1.5%)	0 (0.0%)		0 (0.0%)	1 (1.6%)	
IIIA	26 (36.1%)	18 (25.7%)		26 (38.8%)	18 (24.0%)		22 (27.2%)	22 (36.1%)	
IIIB	16 (22.2%)	26 (37.1%)		14 (20.9%)	28 (37.7%)		29 (35.8%)	13 (21.3%)	
IVA	1 (1.4%)	8 (11.4%)		1 (1.5%)	8 (10.7%)		8 (9.9%)	1 (1.6%)	
IVB	7 (9.7%)	11 (15.7%)		5 (7.5%)	13 (17.3%)		15 (18.5%)	3 (4.9%)	
Median OS (months)	34	11	** <0.001**	37	12	** <0.001**	13	40	** <0.001**

*Alb, albumin; Tbil, total bilirubin; NLR, neutrophil-to-lymphocyte ratio; LMR, lymphocyte-to-monocyte ratio; SII, systemic immune-inflammation index; CA, carbohydrate antigen; LNI, lymph node invasion; AJCC, American Joint Committee on Cancer; OS, overall survival. The bold values represent that P value is less than 0.05*.

**Figure 1:**
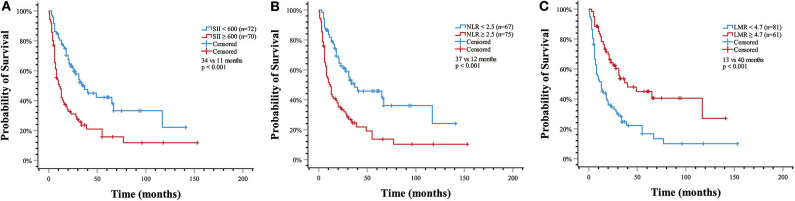
Estimated overall survival with **(A)** systemic immune-inflammation index (SII) >600 (median OS, 11 vs. 34 months, *p* < 0.001); **(B)** neutrophil-to-lymphocyte ratio (NLR) > 2.5 (median OS, 12 vs. 37 months, *p* < 0.001); **(C)** lymphocyte-to-monocyte ratio (LMR) > 4.7 (median OS, 40 vs. 13 months, *p* < 0.001).

Kaplan–Meier analysis demonstrated that SII (11 vs. 34 months, *p* < 0.001) and NLR (12 vs. 37 months, *p* < 0.001) were associated with shorter OS, whereas LMR was associated with higher OS (40 vs. 13 months, *p* < 0.001) (Figure 1).

### Association of Inflammatory Indicators and Surgical Outcomes

We further explored the association between the level of different inflammatory markers and surgical outcome (Table 4). SII and NLR were negative predictors of surgical blood loss, whereas LMR indicated less blood loss. However, there was no significant difference in postoperative length of hospital stay and postoperative complications.

**Table 4:** Association of SII and surgical outcomes.

**Parameter**	**SII**			**NLR**			**LMR**		
	** <600**	**≥600**	***p*-value**	** <2.5**	**≥2.5**	***p*-value**	** <4.7**	**≥4.7**	***p*-value**
	**N = 72**	**N = 70**		**N = 67**	**N = 75**		**N = 81**	**N = 61**	
Mean blood loss (ml)	100 (50–237.5)	200 (50–400)	**0.015**	100 (40–250)	200 (80–400)	**0.004**	200 (100–400)	80 (40–200)	**0.000**
Postoperative complications^*a*^	7 (9.7%)	15 (21.4%)	0.054	7 (10.4%)	15 (20.0%)	0.116	15 (18.5%)	7 (11.5%)	0.251
Median postoperative hospital stay (days)	9 (6–12)	10 (7–15)	0.069	9 (6–12)	10 (7–14)	0.226	10 (7–14)	9 (6–12)	0.097

*SII, systemic immune-inflammation index; NLR, neutrophil-to-lymphocyte ratio; LMR, lymphocyte-to-monocyte ratio. The bold values represent that P value is less than 0.05*.

a *Patients with at least one complication as follows were calculated as having postoperative complications: bile leakage, intraabdominal bleeding, infectious complications, and hepatic failure*.

### Prognostic Factors for OS

Ten variables were included in the univariate analysis. Among them, age, total bilirubin, albumin, SII, NLR, LMR, CA 19-9, and AJCC stage were correlated with OS (Table 5).

**Table 5:** Predictors of overall survival.

**Cox for OS variable^**a**^**	**Univariate analysis**		**Multivariate analysis**^****c****^	
	**HR (95% CI)**	***p*-value**	**HR (95% CI)**	***p*-value**
Age				
<60≥60	1.458 (0.9,424−2.256)	0.090	1.511 (0.957–2.386)	0.077
Sex (male)	0.904 (0.598–1.365)	0.631	–	–
aCCI	0.991 (0.877–1.120)	0.885	–	–
Tbil^*^				
<34≥34	2.820 (1.673–4.752)	** <0.001**	1.073 (0.569–2.022)	0.828
Alb^*^				
<35≥35	0.506 (0.308–0.832)	**0.007**	1.064 (0.579–1.954)	0.842
SII^*^				
<600≥600	2.261 (1.496–3.418)	** <0.001**	1.694 (1.069–2.684)	**0.024**
NLR^*^				
<2.5≥2.5	2.363 (1.551–3.600)	** <0.001**	1.170 (0.607–2.253)	0.639
LMR^*^				
<4.7≥4.7	0.422 (0.274–0.650)	** <0.001**	0.931 (0.509–1.702)	0.817
CA 19-9^*^				
<37≥37	3.648 (2.329–5.713)	** <0.001**	2.407 (1.472–3.933)	** <0.001**
AJCC stage*^b^		** <0.001**		**0.026**
0	<0.001	0.966	<0.001	0.994
I	0.075 (0.018–0.319)	<0.001	0.144 (0.032–0.641)	0.011
II	0.112 (0.038–0.326)	<0.001	0.227 (0.073–0.704)	0.010
III	0.518 (0.322–0.833)	0.007	0.693 (0.419–1.147)	0.154
IV	Indicator		Indicator	

*aCCI, age-adjusted Charlson Comorbidity Index; Tbil, total bilirubin; Alb, albumin; NLR, neutrophil-to-lymphocyte ratio; LMR, lymphocyte-to-monocyte ratio; SII, systemic immune-inflammation index; CA, carbohydrate antigen; AJCC, American Joint Committee on Cancer. The bold values represent that P value is less than 0.05*.

* *indicates variables included in the multivariate model*.

a *The total number of this model is N = 142*.

b *AJCC stage IV (n = 27) was selected as the indicator of the multiclass variable*.

c *C-index of the multivariate model is 0.752*.

**Figure 2:**
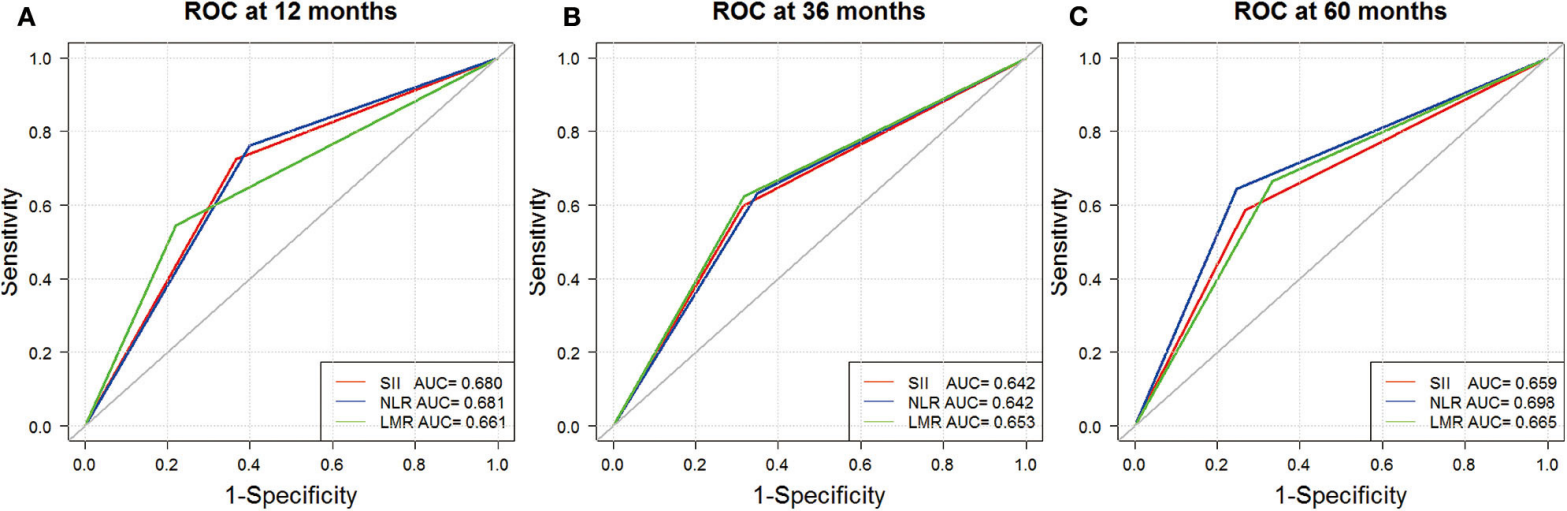
Receiver operating characteristic analyses for SII (red), NLR (blue), and LMR (green) at 12 **(A)**, 36 **(B)**, and 60 months **(C)**.

As illustrated in Table 2, C-index for SII, NLR, and LMR was 0.624, 0.626, and 0.622, respectively. We also evaluated their prognostic value by ROC analysis (Figure 2). The area under the curve (AUC) of SII and NLR was similar at 12 and 36 months, but the AUC of SII was significantly less than that of NLR at 60 months.

In the Cox regression multivariate model of survival, SII ≥600 (*p* = 0.024), CA 19-9 ≥37 U/ml (*p* < 0.001), and higher TNM stage (*p* = 0.026) were independent predictors of shorter OS. Although median OS was comparable between groups as identified by NLR and LMR, they seemed not to be independent predictors (*p* > 0.05). The hazard ratio (HR) was 1.694 (95% confidence interval [CI]: 1.069–2.684) for SII and 2.407 (95% CI: 1.472–3.933) for CA 19-9. As for the TNM stage, with stage IV (*n* = 51) as the indicator, HR was < 0.001 for stage 0, 0.144 (95% CI: 0.032–0.641) for stage I, 0.227 (95% CI: 0.073–0.704) for stage II, and 0.693 (95% CI: 0.419–1.147) for stage III.

### Construction and Validation of Nomogram

**Figure 3:**
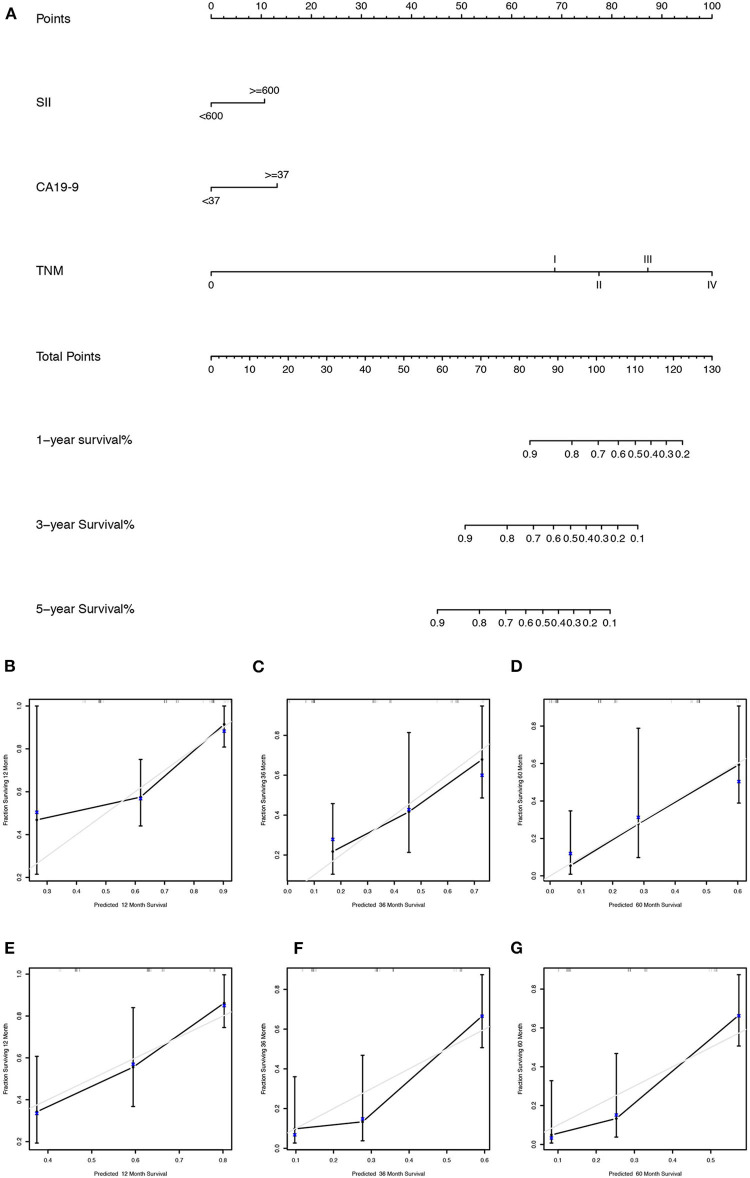
**(A)** The nomogram for predicting 1-, 3-, and 5-year survival probabilities of GBC patients based on SII levels, CA 19-9 levels, and AJCC stage; calibration curves of the nomogram for predicting survival probabilities of 1 **(B)**, 3 **(C)**, and 5 years **(D)** in the training cohort and 1 **(E)**, 3 **(F)**, and 5 years **(G)** in the validation cohort. The x axis plotted nomogram-predicted probabilities, whereas the y axis plotted observed probabilities of OS. The gray diagonal line indicated the ideal calibrated model.

**Figure 4:**
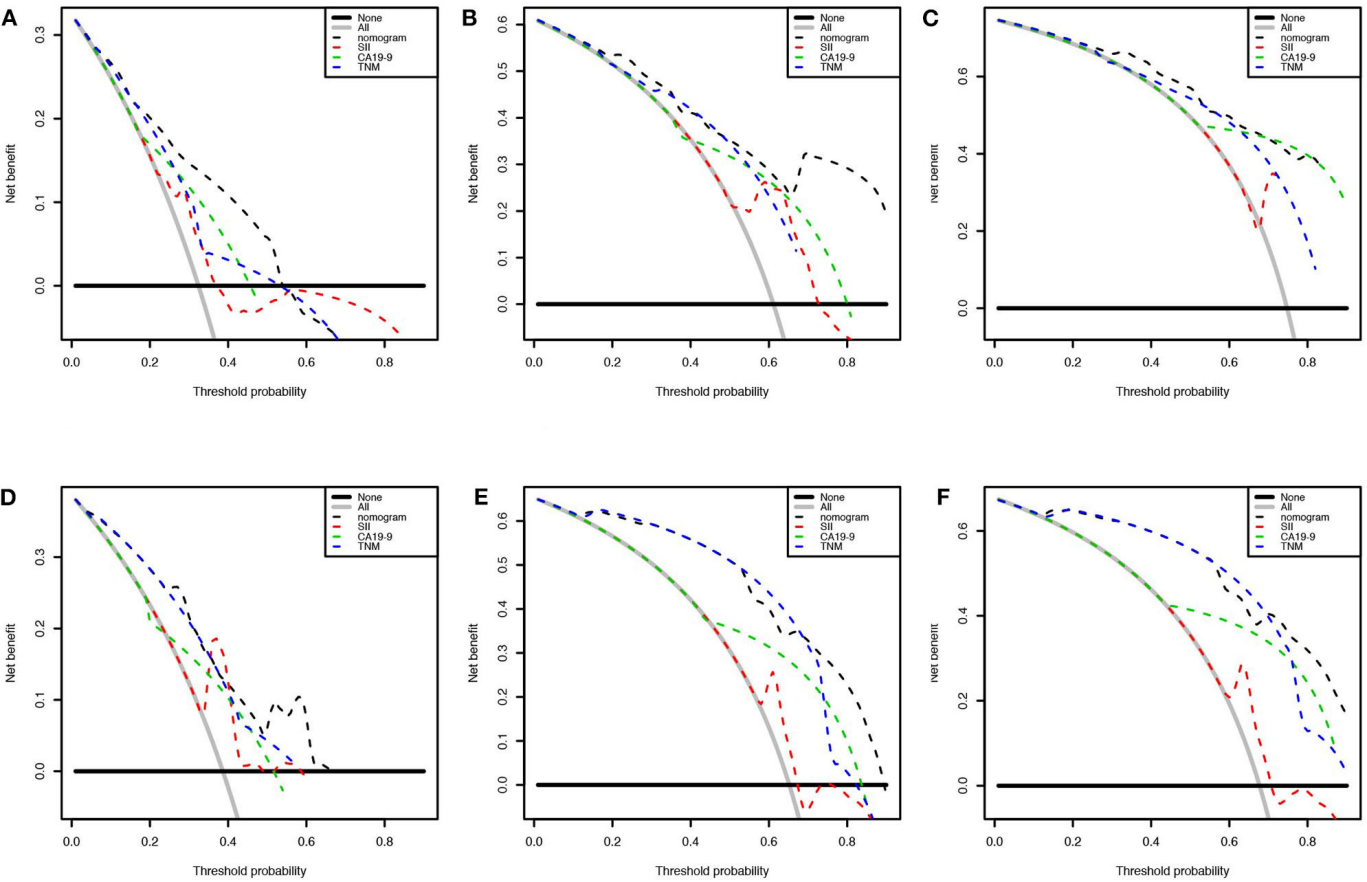
Decision curve analysis presented the clinical net benefit between different models. Nomogram was compared with the AJCC 8th edition stage system, SII, and CA 19-9 of 1 **(A)**, 3 **(B)**, and 5 years **(C)** in the training cohort and 1 **(D)**, 3 **(E)**, and 5 years **(F)** in the validation cohort. The horizontal solid black line represented the assumption without any event. The solid gray line represented the assumption that all patients would experience the event. The dashed line showed the net benefit of models (black: nomogram, red: SII, green: CA 19-9, blue: AJCC 8th edition stage system).

We first randomly equally divided the patients into the training cohort and the validation cohort. The nomogram predicting 1-, 3-, and 5-year survival probabilities was established based on the independent prognostic factors in Table 5 in the training cohort (Figure 3), and the predictive ability of this model was assessed by C-index, which was 0.755 in the training cohort and 0.754 in the validation cohort. The calibration plots adjusted by bootstrapping with 1,000 samples were used to evaluate the performance of the nomogram graphically. These predicted lines overlapped well with reference lines, especially for the 3- and 5-year survival probabilities in the training cohort and the 1-year probability in the validation cohort, which demonstrated good performance of the nomogram. DCA showed that the nomogram had better net benefit than the AJCC staging system, SII, and CA 19-9 alone (Figure 4).

To validate whether SII had a positive effect to the model, we compared the IDI index between the model with or without SII. Further, the results showed that SII could significantly increase the IDI index by 0.042 (*p* = 0.039). Moreover, the model with SII had the best predictive ability with significantly higher IDI and higher C-index than the model substituting SII with NLR (IDI = 0.034, *p* = 0.034, C-index = 0.736) or with LMR (IDI = 0.038, *p* = 0.028, C-index = 0.732). Furthermore, the model combining SII, NLR, LMR, CA 19-9, and TNM staging system did not show superiority to the model with only SII, CA 19-9, and TNM staging system (IDI = −0.006, *p* = 0.727).

### Heterogeneity in the AJCC Stage

**Figure 5:**
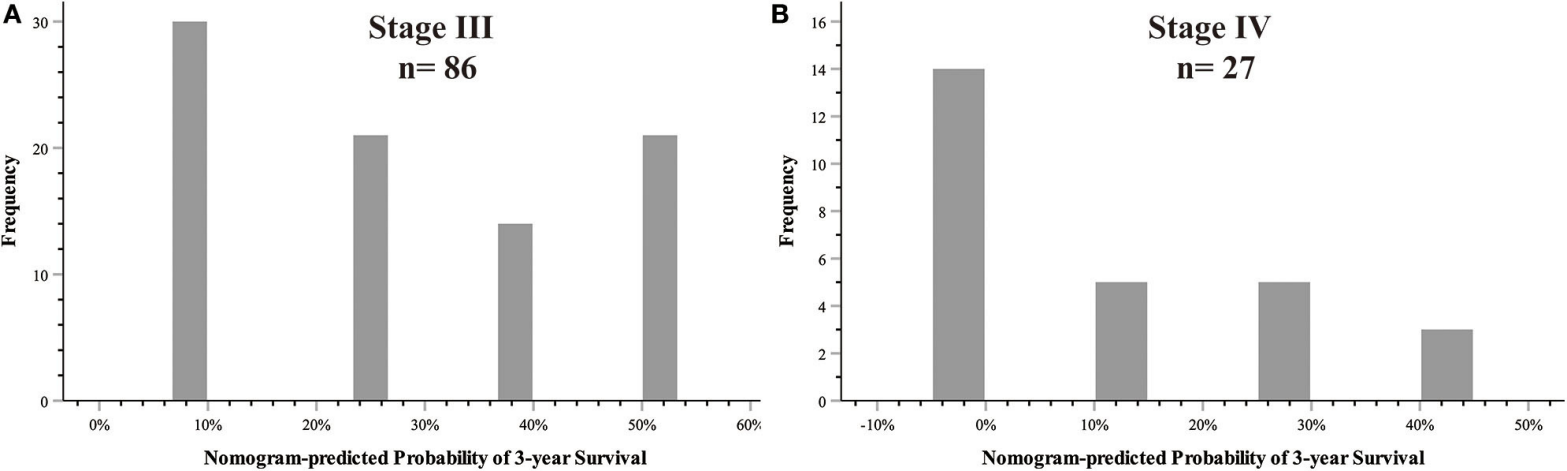
Heterogeneities predicted by nomogram in the AJCC stage. **(A)** AJCC TNM stage III; **(B)** AJCC TNM stage IV.

The 8th AJCC TNM staging system, together with other clinical data, was used to construct the nomogram, and the nomogram showed better discriminatory ability than the AJCC system itself (C-index: 0.755 vs. 0.663 in the training cohort, 0.754 vs. 0.690 in the validation cohort). This indicated that there was heterogeneity even for the same TNM stage, which could be predicted by the nomogram. The nomogram-predicted probability of 3-year survival for stage III (*n* = 86) and stage IV (*n* = 27) is shown in Figure 5.

## Discussion

For GBC, pathology remains the gold standard for diagnosis, and the TNM staging system is widely used for prognostication. However, this system ignores demographic and other clinical features, such as complications and inflammatory status, which could result in heterogeneity.

Inflammatory status is important in tumor development and metastasis (13). NLR, PLR, and LMR are associated with the prognosis of various tumors as inflammatory indicators and are calculated from blood counts owing to their convenience and simplicity. Cho and colleagues reported that NLR and PLR were independent predictors for OS in patients with biliary tract cancer (14). Deng et al. reported that NLR and LMR were independent prognostic factors for OS in patients with GBC, whereas PLR was considered not independent in multivariate analysis (15). In that study, the cut-off point of NLR and LMR was 2.61, and 2.66, respectively. The difference could have been generated by the different populations enrolled, because median LMR was 2.98, whereas it was 4.20 in our study. Besides, the methods for cut-off point determination were different in these two studies. Some researchers have presented different results with these inflammatory markers. Choi and colleagues reported that NLR and PLR were associated with OS in univariate analysis in patients with advanced GBC, but only PLR was independent in multivariate analysis (16). This could have been because some variables, such as PLR in the previous research, were considered more significant in the backward stepwise elimination analysis. Similar results were also seen in studies analyzing these markers, together with other tumors (17, 18).

SII was first described in hepatocellular carcinoma and proved effective as a predictor in several solid tumors (19). SII was calculated from blood counts and somehow combined by PLR and NLR. SII has been demonstrated to have better predictive ability for OS than NLR and PLR for patients with pancreatic ductal adenocarcinoma (10). However, as far we know, ours is the first study of baseline SII as a negative prognostic factor in GBC.

There are contradictory results as to whether baseline pretreatment SII can predict clinical outcome for pancreatic ductal adenocarcinoma (9, 20). Our study demonstrated that NLR and SII were predictors of poor OS in GBC patients, whereas LMR was a protective prognostic factor in univariate analysis. A similar association was found between these markers and surgical blood loss. In our study, SII, NLR, and LMR were all associated with albumin, CA 19-9, pathological features, and surgical blood loss, and their C-index was similar. Besides, the AUC of SII and NLR was similar at 12 and 36 months, whereas the AUC at 5 years suggested that NLR had better prognostic value than SII had. This indicates that NLR has a greater long-term prognostic value, which should be evaluated by further large-scale studies, because in the present study, only 19 patients lived >5 years after surgery. Meanwhile, the multivariate analysis showed that SII was a superior predictor of OS, which was the only independent one of these inflammatory markers.

SII and LMR were calculated from the numbers of platelets, neutrophils, lymphocytes, and monocytes, which could represent the majority of blood cell types. Our study eventually excluded LMR by backward stepwise analysis, but it did not mean that monocytes were not important in tumorigenesis. Monocytes can be induced into macrophages and polarized into different subtypes. The level of peripheral monocytes is associated with tumor-associated macrophages (TAMs) (21). TAMs are related to tissue remodeling, immunoregulation, and angiogenesis, which can enhance tumor cell migration, and immune evasion (22). As discussed previously, elimination of LMR was eliminated because of statistical significance, while more and larger studies are needed.

Our study aimed to incorporate the demographic and other clinical features into the AJCC 8th edition staging system to construct a survival model for GBC patients. Figure 5 shows that there was heterogeneity in the same TNM stage, especially for high-level GBC, which could be figured out by the nomogram. Nomograms are progressively being used for estimating prognosis and personalized medicine. This graphical tool could be convenient for doctors in the clinic when the parameters are all common laboratory tests. Our nomogram showed that SII ≥ 600 weighed 10.66, CA 19-9 ≥ 37 U/ml weighed 13.15, TNM stage I weighed 68.61, TNM stage II weighed 77.42, TNM stage III weighed 87.19, and TNM stage IV weighed 100. Although SII and CA 19-9 contributed little in terms of nomogram scores compared with TNM stage, they weighed more than the difference between the adjacent TNM stages when it was at least stage I. Apparently they could be of assistance in precise prognostication of survival, especially in high-stage GBC patients. This advantage was also shown on DCA curves. In the 1-year DCA curve in the training cohort, the greatest net benefit between nomogram and TNM stage was generated when the threshold probability was ~0.35. It could be calculated for a score of about 87 in the nomogram, which was close to the weight of TNM stage III (87.19).

The present study had several limitations. First, it was based on retrospective data from a single institution in one region. Second, the entire cohort was used to build the nomogram, and we did not have an internal validation cohort because of the small population size. Third, although laboratory data were all collected before surgery, the timing of blood withdrawal was variable. Finally, because all patients enrolled underwent surgical resection, there could be some patients with advanced GBC who were not eligible for curative surgical resection and gave up for further treatment. Further prospective studies with external validation are needed in the future.

This study suggests that baseline pre-operative SII is an independent negative prognostic indicator for survival of patients with GBC. Our training cohort generated a nomogram based on SII and the AJCC 8th edition staging system, which could serve as a useful prognostic tool for GBC patients.

## Data Availability Statement

The raw data supporting the conclusions of this article will be made available by the authors, without undue reservation.

## Ethics Statement

The studies involving human participants were reviewed and approved by the Ethics Committee of Peking Union Medical College. The patients/participants provided their written informed consent to participate in this study.

## Author Contributions

YM and HY: study concepts. LS and YJ: study design. WH and MZ: data acquisition. LS and XL: quality control of data and algorithms. YJ and YX: data analysis and interpretation. YJ and HZ: statistical analysis. WH and HX: manuscript preparation. SZ and BJ: manuscript editing. XS and SD: manuscript review. All authors contributed to the article and approved the submitted version.

## Conflict of Interest

The authors declare that the research was conducted in the absence of any commercial or financial relationships that could be construed as a potential conflict of interest.

## Abbreviations

SII: systemic immune-inflammation index
GBC: gallbladder carcinoma
AJCC: American Joint Committee on Cancer
NLR: neutrophil-to-lymphocyte ratio
LMR: lymphocyte-to-monocyte ratio
PLR: platelet-to-lymphocyte
OS: overall survival
aCCI: age-adjusted Charlson Comorbidity Index
CA 19-9: carbohydrate antigen 19-9
IQR: interquartile range
C-index: Harrell's concordance index
ROC: receiver operating characteristic
DCA: decision curve analysis
AUC: area under the curve
HR: hazard ratio
CI: confidence interval
TAMs: tumor-associated macrophages.
